# Distinct microbial communities among different tissues of citrus tree *Citrus reticulata* cv. Chachiensis

**DOI:** 10.1038/s41598-020-62991-z

**Published:** 2020-04-08

**Authors:** Yongxian Wu, Mengqiu Qu, Xinhua Pu, Jintian Lin, Benshui Shu

**Affiliations:** Guangzhou City Key Laboratory of Subtropical Fruit Trees Outbreak Control, Zhongkai University of Agriculture and Engineering, Guangzhou, China

**Keywords:** Microbial ecology, Microbiome

## Abstract

Plant microbiota colonize all organs of a plant and play crucial roles including supplying nutrients to plants, stimulating seed germination, promoting plant growth, and defending plants against biotic and abiotic stress. Because of the economic importance, interactions between citrus and microbes have been studied relatively extensively, especially citrus-pathogen interactions. However, the spatial distribution of microbial taxa in citrus trees remains under-studied. In this study, *Citrus reticulata* cv. Chachiensis was examined for the spatial distribution of microbes by sequencing 16S rRNA genes. More than 2.5 million sequences were obtained from 60 samples collected from soil, roots, leaves, and phloem. The dominant microbial phyla from all samples were Proteobacteria, Actinobacteria and Acidobacteria. The composition and structure of microbial communities in different samples were analyzed by PCoA, CAP, Anosim and MRPP methods. Variation in microbial species between samples were analyzed and the indicator microbes of each sample group were identified. Our results suggested that the microbial communities from different tissues varied significantly and the microenvironments of tree tissues could affect the composition of its microbial community.

## Introduction

Plants host diverse microbes that colonize to, on and in their tissues^1^. Based on their habitats, plant-associated microbial communities are referred to as rhizosphere microbiome, rhizoplane microbiome, phyllospher microbiome, and endosphere microbiome, respectively^2–5^. Different microbiomes interact with host plants and impact plants in various ways. In general, plant-associated microbial organisms can potentially have both positive and negative impacts on plant growth, development, and health^1^. Direct impacts on plant growth and development by microorganisms include improved nutrient accessibility such as nitrogen fixation and phosphate solubilization; altered microenvironments such as changed acidity (pH); and hormonal stimulation (phytohormone production)^3,6^. Microorganisms are also involved in the promotion or suppression of plant diseases either directly (such as antibiotics production) or indirectly (via disease resistance)^7^. Accordingly, characterization of microbial compositions and dynamics in different plant tissues and elucidation of functions associated with specific microbes or microbial communities will provide the basis for developing commercially probiotics for plants or designing strategies to manipulate microbes or microbial communities for economic or environmental benefits^8–10^.

Recent advances on culture-independent, high-throughput sequencing of the variable regions of 16 S rRNA genes in microbes and total genome assembly from metagenomic sequence reads has revolutionized our research on the structures and diversity of microbiomes in different plant tissues. As a result, microbiome data on various types of plants have been accumulated rapidly in recent years. Two independent studies on the microbial community in the root of the model plant *Arabidopsis thaliana* revealed consistent results on the core microbiome, with *Actinobacteria* and a few families from *Proteobacteria* enriched consistently in the endosphere compared with rhizosphere^11,12^. Microbiomes from various tissues of many crops such as sugarcane, rice, tomato, maize, sorghum, soybean, and cotton have been characterized to various degrees^13–19^. Microbiomes from certain tissues of fruits and trees including pear, banana, and apple have initially analyzed as well^20–22^.

Orange (*Citrus* × *sinensis*) is one of the most important fruits for humans worldwide. In Brazil alone, 35.6 million tons of oranges were produced based on the most recently available data in 2013. Like other crops and fruits, citrus production faces many hurdles and challenges that need to be overcome to achieve high yield and high quality of oranges. Characterization of microbiomes in different tissues of citrus trees may yield useful information for the improvement of orange production. There is fragmented information available on microbiomes of citrus trees^23^. Most previous investigation on citrus-associated microbiomes are analyses on huanglongbing-infected, or specially-treated samples^24–29^.

*Citrus reticulata* cv. Chachiensis is an important citrus variety widely cultivated around the world and the orange peel of the fruit was regarded as the valuable material which could be processed as food, tea drinks, and seasoning^30^. Besides, the orange peel owned antioxidant properties and extremely high medicinal value was also used as herb-medicine^31^. In China, *C. reticulata* cv. Chachiensis has grown in Xinhui District, Jiangmen City, Guangdong Province was considered as one of the most important cash crops for locals and the orange peel named genuine *Pericarpium Citri Reticulatae* (Guang Chen Pi) was admired as the best Chen pi which possesses the excellent clinical efficacy^32^. Plant-related microorganisms could protect the host from pathogens and pests, help plants use nutrients, and improve plant stress resistance^33^, while the microbial community of *C. reticulata* cv. Chachiensis remains unknown. In this study, endosphere microbiomes in soils, roots, leaves, and phloem of *C. reticulata* cv. Chachiensis were systematically investigated by sequencing 16S rRNA genes. The composition and structure of the microbial community in different tissues were performed by PCoA, CAP, Anosim and MRPP methods. Variation in microbial compositions among citrus tissues were analyzed and indicator microbes for different samples were identified. Our results suggested that the microbial community from different tissues of *C. reticulata* cv. Chachiensis in south china varied significantly. Our results laid a foundation for future studies on microbial communities on citrus and their implications on orange production.

## Results

### Distribution of 16s sequences among samples

More than 2.5 million sequence reads were obtained from 60 samples, with 15 samples from each type of the following sources: soils, roots, leaves, and phloem. All the raw data of samples were submitted to the SRA database with the submission number of SUB6069689. Specifically, 639,200 (24.90%) were from soils, 688,345 (26.81%) from roots, 639,373 (24.91%) from leaves, and 599,685 (23.4%) from phloem. Among the total sequence reads, 1,454,442 (56.7%) were assigned to chloroplast and mitochondrial reads, 1,085,802 (42.3%) assigned to other classifiable reads, and 26,359 (1.0%) were unclassifiable reads. Among the chloroplast and mitochondrial reads, 42.81%, 37.09%, 19.89%, and 0.21% were from leaf, phloem, root, and soil samples, respectively. Most other classifiable reads, 57.00%, and 36.17%, respectively, were from soil and root samples, and only 1.39 and 5.44% were from leaf and phloem samples (Tables 1 and S1).

**Table 1:** Summary of the sequencing data.

Samples	sequence reads		classifiable reads		chloroplast and mitochondrial reads	
	number	Percentage (%)	number	Percentage (%)	number	Percentage (%)
Soils	639200	24.90	618955	57.00	2985	0.21
Roots	688345	23.36	392682	36.17	289310	19.89
Phloem	599685	26.81	59069	5.44	539381	37.09
Leaves	639373	24.91	15096	1.39	622766	42.81
Total	2566603	100	1085802	100	1454442	100

### Distribution of OTUs

**Figure 1:**
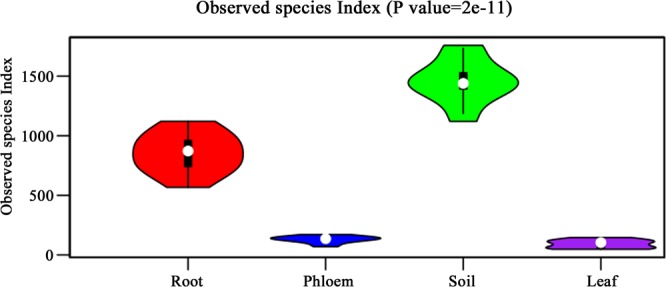
The measurable OUT number of microbial species from different tissues of citrus trees. R stands for root samples, P stands for phloem samples, S stands for soil samples and L stands for leaf samples.

After excluding chloroflexi and mitochondrial reads and unclassifiable unique reads, the remaining reads were assembled into OTUs. The distribution of total OTUs were 4733, 3520, 421, and 583, respectively, for soil, root, leaf, and phloem samples. Based on our technical reproducibility, we set the criterion for ‘measurable OTUs’ as ≥20 reads in at least three samples of the same type. Based on this criterion, the measurable OTUs were 2245, 1034, 55, and 111 for soil, root, leaf, and phloem samples (Fig. 1).

### β diversity analysis of samples

**Figure 2:**
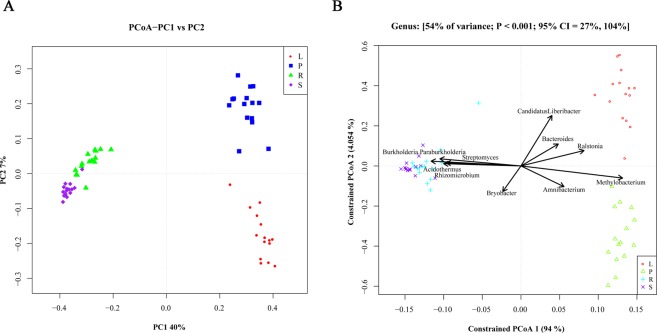
The results of PCoA and CAP analyses in all the samples. (**A**) The PCoA analysis of microbial species among the total samples. The abscissa (x) represents the first principal component, the percentage represents the contribution of the first principal component to the sample difference; the ordinate (y) represents the second principal component, and the percentage represents the contribution of the second principal component to the sample difference. Each point in the figure represents a sample, and samples of the same group are represented by the same color. (**B**) The CAP analysis of microbial species among the total samples. The numbers at the top of the figure represent the variance contribution of these factors, P value, and the confidence interval (95% CI) of the variability, respectively. Species with a higher degree of association with the group are indicated by arrows. The length of the line represents the degree of correlation between an environmental factor and the distribution of the community and the distribution of the species. The longer the arrow, the greater the correlation.

Principal co-ordinates analysis (PCoA) of samples from different parts of citrus trees were performed. The samples with the closer distance of Unweighted Unifrac indicated higher similarity between microbial communities. As shown in Fig. 2A, the microbial community in soil samples was more similar to that in root samples with all samples clustered together. The microbial communities in leaf and phloem samples differed significantly. The microbial communities differed significantly among the groups of samples from leaves and phloem. Canonical analysis of principal coordinates (CAP) yielded results similar to PCoA results. CAP analyses might reflect sample-to-sample variation between different sample groups, and the results indicated that all samples were divided into three clusters: 1) soil and root, 2) leaf, 3) phloem (Fig. 2B). The compositions of microbiota among different samples correlated among groups, with major species including *Burkholderia Paraburkholderia*, *Streptomyces*, *Bryobacter*, *Acidothermus* and *Rhizomicrobium* for the soil and root groups; *Methylobacterium* and *Amnibacterium* for phloem groups; and *Ralstonia*, *Bacteroides*, and *Candidatus Liberibacter* for leaf groups (Table 2).

**Table 2:** Genus with a higher degree of correlation in different groups.

Genus	group
*Burkholderia.Paraburkholderia*	R,S
*Streptomyces*	R,S
*Bryobacter*	R,S
*Methylobacterium*	P
*Amnibacterium*	P
*Acidothermus*	R,S
*Rhizomicrobium*	R,S
*Ralstonia*	L
*Bacteroides*	L
*CandidatusLiberibacter*	L

R: Roots, S: Soils, P: Phloem, L: Leaves.

### Core microbial communities

**Figure 3:**
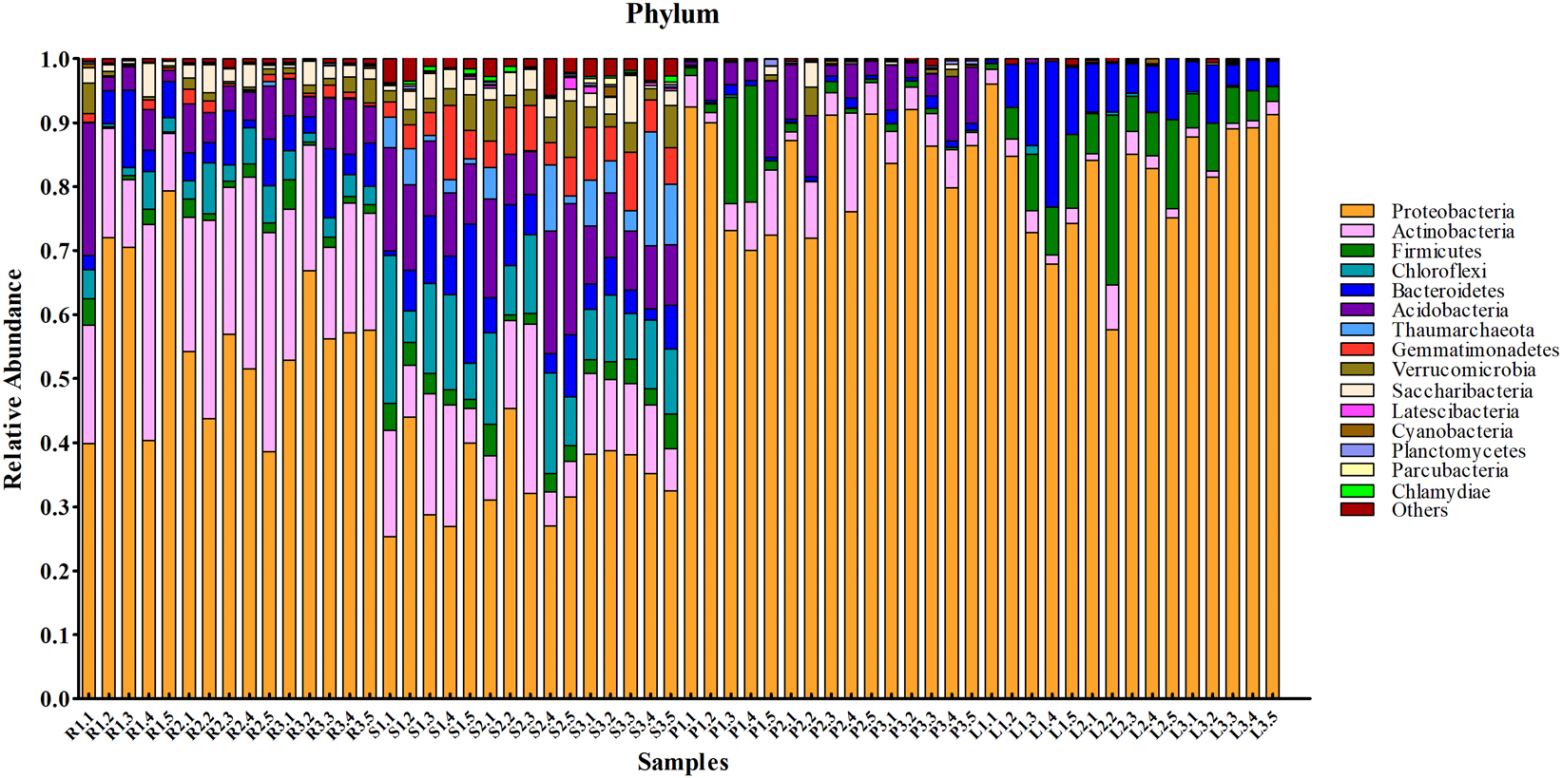
The core phylum of microbial communities in different tissues of citrus trees.

Twenty-three phyla of microbes were identified from all samples. The dominant phyla were *Proteobacteria*, *Actinobacteria*, and *Acidobacteria*. The core microbial community, defined as the shared microbial species among all independent samples within the same group, is shown in Fig. 3. Ten of the most abundant microbial species in the root and soil groups were from *Proteobacteria*, *Actinobacteria*, *Acidobacteria*, *Bacteroidetes*, *Firmicutes*, *Chloroflexi*, *Gemmatimonadetes*, *Verrucomicrobia*, *Saccharibacteria*, and *Thaumarchaeota*. However, the proportion of microbes from different phyla showed significant differences between these two groups. For the phloem groups, the major phyla were similar to those from root and soil groups except for *Gemmatimonadetes*. Core microbial species in the leaf group were *Proteobacteria*, *Actinobacteria*, *Acidobacteria*, *Bacteroidetes*, *Firmicutes*, *Chloroflexi*, *Cyanobacteria*, and *Fusobacteria*. *Proteobacteria* contained the majority of microbial species in both the phloem and leaf samples, representing more than 80% of identified species. The core families and genus of microbial communities were shown in Supplemental Fig. 1.

### Similarity and difference among microbial community structures

The analytical methods Anosim and Multi-Response Permutation Procedure (MRPP) were used to compare potential similarities and differences among community structures of different sample groups. ANOSIM analyses (analysis of similarities) revealed that all R-values were greater than 0 and all P-values were 0.001, indicating significant differences between microbial community structures among different sample groups (Table 3). MRPP analyses also revealed significant differences with values of expecting delta ranged from 0.08305 to 0.3433 and values of significance 0.001 (Table 4).

**Table 3:** Inter-group difference analyzed by Anosim.

Group	R-value	P-value
R-S	1	0.001
R-P	0.6731	0.001
L-S	0.9996	0.001
L-P	1	0.001
L-R	0.983	0.001

R: Roots, S: Soils, P: Phloem, L: Leaves.

**Table 4:** Inter-group difference analyzed by MRPP.

Group	A:	observed-delta	expected-delta	Significance
P-L	0.3433	0.4073	0.6202	0.001
R-P	0.3112	0.5149	0.7475	0.001
R-L	0.2567	0.5663	0.7619	0.001
P-S	0.3273	0.5052	0.751	0.001
S-L	0.2821	0.5566	0.7754	0.001
R-S	0.08305	0.6642	0.7244	0.001

R: Roots, S: Soils, P: Phloem, L: Leaves.

### Microbial species analyses

The metastat software based on Fisher exact test was used to analyze the differences in microbial species between different sample groups. The species with significant differences between different groups were filtered out according to the P and Q values (Table S2). *Rhodopila* was unique in the phloem group. *Halomonas* and *Ralstonia* were enriched in the leaf and phloem groups (*P* < *0.01*). The abundance of these two microbes in the leaf group was significantly higher than that in the phloem group (*P* < *0.01*). *Methylobacterium* and *Sphingomonas* were also very abundant in both the leaf and phloem groups (*P* < *0.01*), with higher abundance in the phloem group. *Streptomyces*, *Burkholderia*-*Paraburkholderia*, and *Acidibacter* were mainly in the root and soil groups with higher abundance in the root group between the two (*P* < *0.05*). *Nitrosopumilus* was enriched in the soil group compared to the other three groups (*P* < *0.01*). *Rhizobium* was more abundant in the root group (*P* < *0.01*).

### Identification of microbial indicators

**Figure 4:**
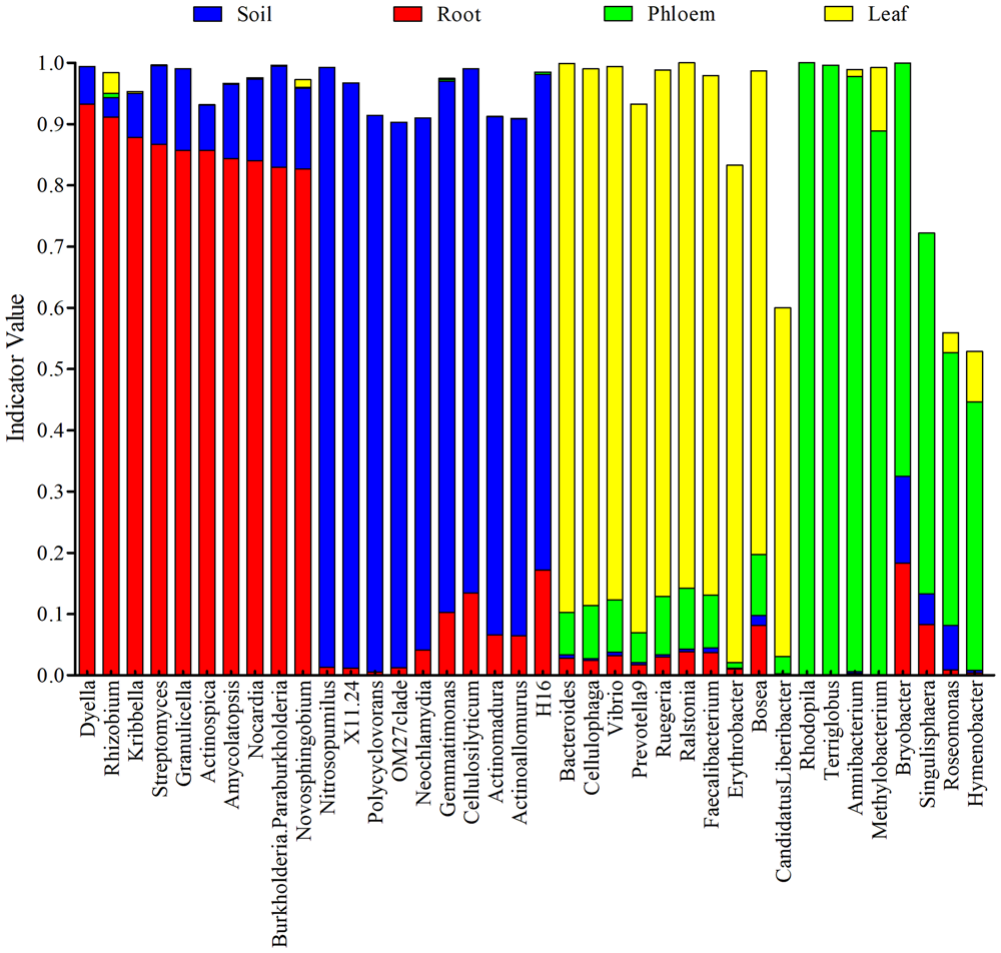
The microbial indicators and indicator-values in different tissues of citrus trees. Different colors in the figure represent different groups. The indicator value of a species in a single group ranges from 0 to 1. When the indicator value is closer to 0, the species classification is almost non-existent in the group; while is closer to 1 indicates that the species is unique to the group and exists in almost all samples.

Indicator-values were used as criteria to access the uniqueness of microbial species in each group. As shown in Fig. 4, microbes specific to different groups were found. Among the unique microbes, *Dyella*, *Rhizobium*, *Kribbella*, *Streptomyces*, *Granulicella*, *Actinospica*, *Amycolatopsis*, *Nocardia*, *Burkholderia*. *Paraburkholderia* and *Novosphingobium* were identified as the indicators for the root group; *Nitrosopumilus*, *X11*.24, *Polycyclovorans*, *OM27clade*, *Neochlamydia*, *Gemmatimonas*, *Cellulosilyticum*, *Actinomadura*, *Actinoallomurus* and H16 were indicators for the soil group. *Bacteroides*, *Cellulophaga*, *Vibrio*, *Prevotella9*, *Ruegeria*, *Ralstonia*, *Faecalibacterium*, *Erythrobacter*, *Bosea*, and *Candidatus Liberibacter* were indicators for the leaf group; and *Rhodopila*, *Terriglobus*, *Amnibacterium*, *Methylobacterium*, *Bryobacter*, *Singulisphaera*, *Roseomonas*, and *Hymenobacter* were indicators for the phloem group.

## Discussion

Holobionts with high diversity residing in different plant tissues are regarded as plant microbiota^1^. The structure and function of microbial communities have attracted a great deal of attention because of increasing evidence suggesting critical roles of microbiomes in plant development and survival^34^. Plant-associated microorganisms could directly provide nutrition to plants via nitrogen fixation and phosphate solubilization^35^. Microbes can also induce resistance to other biotic and abiotic stresses through producing or degrading phytohormone^35^. Hence, it is necessary to investigate local microbial communities associated with plants to examine their impact on various aspects of a given plant species. Different plants host different microbial communities. Meanwhile, the microbial communities of plants could be affected by many factors. For example, the bacterium ‘*Candidatus* Liberibacter asiaticus’ (CLas) which induced the citrus devastating disease named Huanglongbing (HLB) could alter the root microbial community structure of *Citrus limon* and *Citrus sinensis*^36^. Administration of antibiotics including penicillin disrupted the interspecies microbiological connections and induced major changes in root bacterial community structure of grapefruit trees (*Citrus paradise* Macf.)^37^. Besides, the diversity of fungal endophyte communities in leaf, stem, trunk, and root tissues of *C. reticulata* cv. Siyahoo was observed^38^. However, a high similarity of dominant bacterial communities of *Pericarpium Citri Reticulatae* ‘Chachiensis’ obtained from different orchards in Xinhui District was observed^39^. In order to assess differences and dynamic changes in microbial communities among different tissues of *C. reticulata* cv. Chachiensis, 16S rRNA gene sequencing has been adapted to analyze microbes in samples derived from nearby soil, roots, leaves, and phloem. Our results demonstrated that the numbers and types of OTUs varied greatly among different sample groups. Among the microbes identified in this study, some genera have been well studied in the past few decades, whereas others were novel and unique. Identification of these microbes will facilitate comparative research on known microbes and functional studies on unique species. Furthermore, the microbial communities of many plant species were varied by geographical location. For example, the microbial diversity of *C. reticulate blanco* var. clementine in different regions was confirmed by 16S rDNA fingerprinting analysis^40^. For spruce (*Picea* spp.) trees, a significant difference of the microbial taxonomical composition in the phyllosphere was observed at three locations^41^. Moreover, the microbial community structure of malts from different cropping zones was variable. Among them, the effects of geographical location on the fungal community were more obvious than bacterial community^42^. Therefore, to study the microbial community of *C. reticulata* cv. Chachiensis more systematically, the effect of different geographical locations should be concerned and further studied.

Large differences in community structures in different microbiota have been observed in other plants from previous studies^14,43^. Microenvironments of tree tissues are likely one of the major factors driving alteration in the composition of microbial communities^44^. Microenvironments in tissues of citrus trees might also play a major role in the variation of microbial communities observed here. As an open system, plants constantly obtain minerals and water from the surrounding soil. In plant-microbe interactions, a plant may play an active role in shaping its associated microbial communities based on its needs. In turn, microbes may also affect plant physiology. The dynamic interactions among plants, microbes, and other environmental factors determine the functioning of an ecological system^45–47^. Soil by far harbors the most biodiversity and is the largest reservoir of microbes. Soil microbes impact plant microbiota profoundly^48^. Roots of a plant are the initial and main sites for plant-microbe intimate interactions. Rhizoplane, one of the root-associated layers, serves as a critical gate that regulates microbial entry into roots^49^. A comprehensive analysis indicated that no difference existed in the microbial community structure of rhizosphere and associated bulk soil samples collected from twelve citrus varieties which distributed on six continents^50^. In this study, the microbial community structure in roots was also similar to that in soil, suggesting that root-associated microbes were mainly derived from the soil biome^35^. Alternatively, the exudates containing sugars, organic acids and amino acids secreted by plant roots have strongly affected the composition of microbes in surrounding soil. *Proteobacteria*, *Actinobacteria*, *Acidobacteria* and *Bacteroidetes* have been reported as the primary consumers of plant exudates and the predominant taxa of citrus^50,51^. Other reports supported the above results and the microbial community of other plants including maize, arabidopsis and tamarix consists of a few dominant phyla, such as *Proteobacteria*, *Actinobacteria*, and *Bacteroidetes*^16,52,53^. In this study, *Proteobacteria*, *Actinobacteria*, *Acidobacteria*, *Bacteroidetes*, and *Firmicutes* were ranked as the top five core microbes in soil and root samples, which was similar to the global citrus rhizosphere microbiome^50^. However, the core genera of global citrus rhizosphere microbiome including *Pseudomonas*, *Agrobacterium*, *Cupriavidus*, *Bradyrhizobium*, *Rhizobium*, *Mesorhizobium*, *Burkholderia*, *Cellvibrio*, *Sphingomonas*, *Variovorax* and *Paraburkholderia* differed with our results, and the different growth area, seasonal variation and environmental factors could be the reasons for the difference. Furthermore, the genus from these phyla including *Dyella*, *Rhizobium*, *Kribbella*, *Streptomyces*, *Granulicella*, *Actinospica*, *Amycolatopsis*, *Nocardia*, *Burkholderia*. *Paraburkholderia* and *Novosphingobium* were identified as the indicator microbes in the root group. These indicator microbes could play crucial functions to citrus trees, for example, supplying nutrients, conferring resistance against pathogens and parasites^47^.

The microbial communities in citrus leaves and phloem are far smaller in comparison with those in roots and soil in terms of the number of OUTs in each group based on our results. The significant differences could be related to microenvironments in different tissues. Microbes can be originated from different sources, including aerosols or dust^45^. Insects are another important source for plants to gain microbes, including viruses, phytoplasmas, fungi, and bacteria, which are commonly transferred by insects. For example, *Diaphorina citri* Kuwayama (Hemiptera) transmit the bacterium *Candidatus* Liberibacter asiaticus (CLas), which causes a destructive disease called Huanglongbing^54^. Therefore, different sources for plant tissues to gain microbes could be another reason for large differences in their microbial communities. As ground material, the central bacterial community in *Pericarpium Citri Reticulatae* ‘Chachiensis’ separated from the fresh fruit of *C. reticulata* cv. Chachiensis were investigated and Chloroplast and mitochondria were identified as the most abundant bacterial phylum^39^. The significant differences that occurred between our results and the results obtained from *Pericarpium Citri Reticulatae* ‘Chachiensis’ might due to the massive mitochondria and chloroplast of *C. reticulata* existed during the processes of microbes enrichment and DNA extraction^39^. In our knowledge, this is the first time to investigate the microbial community of citrus phloem, which could helpful for understanding the distribution of microbial communities in entire citrus plants. Besides, *Methylobacterium* and *Amnibacterium* were identified as the major genus in citrus phloem. *Methylobacterium* was demonstrated to reduce the proliferation of citrus pathogens including CLas^55^, while the function of *Amnibacterium* should be explained in further study. For the leaf samples, *Proteobacteria*, *Firmicutes* and *Bacteroidetes* were ranked as the top three phyla and counted for more than 96.9% of all the microorganisms. Moreover, *Alphaproteobacteria*, *Betaproteobacteria*, and *Gammaproteobacteria* were the most abundant bacterial classes in leaf samples, which was similar to the results of microbial communities of leaves collected from citrus trees across Florida^29^. Furthermore, some microbes with uniqueness were specific to tissue and were taken as the indicators of that tissue. For example, *Methylobacterium*, *Amnibacterium*, *Rhodopila*, and *Terriglobus* are indicators for the phloem group. *Ralstonia*, *Bacteroides* and *Prevotella* are indicators for the leaf group. The roles of these indicator microbes remain to be clarified.

## Conclusion

In this study, the microbes in different parts of citrus trees were investigated by sequencing 16S rRNA genes. More than 2.5 million sequence reads were obtained from 60 samples and were assembled into OTUs. In total, 4733, 3520, 421, and 583 OTUs, respectively, were identified in samples from soil, roots, leaves, and phloem. The dominant microbial phyla of all samples from citrus trees were *Proteobacteria*, *Actinobacteria*, and *Acidobacteria*. The composition and structure of microbial communities in different plant tissues were analyzed using PCoA, CAP, Anosim and MRPP methods, and the species with a significant difference between groups were identified according to the P and Q values. Our results indicated that the microbial community in different groups were heterogeneous and complex. Indicator microbes for each group were identified based on their uniqueness among different sample groups. The microbial communities in different parts of citrus trees revealed in this study laid a foundation for future studies on microbial diversity and impact on citrus trees.

## Materials and methods

### Sampling sites and collection

The citrus orchard for sampling in this study was in Xinhui, Guangdong Province, China (22°47′N, 113°03′E). *C. reticulata* cv. Chachiensis was planted in this orchard with strict water and fertilizer management and pest control for three years. A total of 60 samples were obtained from citrus tissues including roots, leaves, and phloem as well as surrounding soil. Fifteen samples were obtained from each tissue, and samples from each tissue were referred to as a group. Samples were collected in the spring (March 28) of 2017. Fresh leaf and phloem samples were obtained from trees selected randomly. After collection, samples were immediately frozen in liquid nitrogen and stored at −80 °C. For root samples, roots were cut from trees and washed with sterilized water to remove the sediment for 5 times. After removing water with paper towels, roots were frozen liquid nitrogen. Soil was collected from 3–5 cm underground and immediately frozen in liquid nitrogen.

### DNA extraction and PCR amplification

Citrus tissues were ground with liquid nitrogen to powder for DNA extraction. DNA was isolated and purified using an E.Z.N.A. ^®^Stool DNA Kit (Omega Bio-tek, Norcross, GA, U.S.) according to the manufacturer’s protocols^56^. The DNA quality and concentration were checked on a 1.0% agarose gel after electrophoresis and a NanoDrop ND-2000 spectrophotometer (Thermo Fisher Scientific, United States), respectively. For the amplicon library preparation, DNA was used as the template and the amplification of 16 S rRNA genes was performed by an amplified method^57^ of prokaryotic 16 S rDNA V4-V5 region with the following primers 515 F (5′-GTGCCAGCMGCCGCGG-3′) and 907 R (5′-CCGTCAATTCMTTTRAGTTT-3′), where barcode is an eight-base sequence unique to each sample. PCR reactions were performed in a 20 μL solution containing 4 μL of 5× FastPfu Buffer, 2 μL of 2.5 mM dNTPs, 0.8 μL of each primer (5 μM), 0.4 μL of FastPfu Polymerase, and 10 ng of template DNA. The reaction was run under a program with one cycle at 95 °C for 5 min, followed by 27 cycles at 95 °C for 30 s, 55 °C for 30 s, and 72 °C for 45 s, and a final extension at 72 °C for 5 min.

### Amplicon purification and high-throughput sequencing

After electrophoresis, PCR fragments were extracted from 2% agarose gels using an AxyPrep DNA Gel Extraction Kit (Axygen Biosciences, Union City, CA, U.S.) according to the manufacturer’s instructions. The purified DNA fragments were quantified using QuantiFluor: trademark: -ST (Promega, U.S.). Purified 16 S rDNA amplicons were pooled in equimolar and paired-end sequenced (2 × 250) on HiSeq 2500 platform according to the standard protocol^58^. The sequencing was performed by Health Time Gene Institute in Shenzhen city, China.

### Bioinformatics analysis

After removing the adaptors, primers and low-quality reads, the pair-end reads were assembled into final sequences based on overlapping alignments. The criterion for overlapping was at least 10 bp overlap with a mismatch ratio of less than 0.2. Chimera tags were filtered out using the Gold database by UCHIME (version 4.2.40)^59^. Operational taxonomic unit (OTU) analysis was performed using the Uparse package (version 7.0.1001) with a 97% sequence identity^60^. Each OTU was taxonomically assigned based on the silva database using the RDP classifier^61^. OTUs matched to chloroplast sequences, chondriosome sequences, and unclassified sequences were removed. Only those OTUs with relative abundance > 0.001% (above three tags in at least one sample) in at least one sample were retained. Similarities and differences among microbial communities from different groups were analyzed by principal co-ordinates analysis based on the distance of Unweighted Unifrac^62^. Canonical analysis of principal coordinates was chosen for diversity analyses among different groups^63^. Differences in microbial community structures between groups were examined with the methods of Anosim and Multi Response Permutation Procedure^64^. Differences in microbial species between groups were identified using metastat software based on the Fisher exact test^65^.

## Supplementary information

Supplementary Information.
