# Supplementary material for: Distinct microbial communities among different tissues of citrus tree Citrus reticulata cv. Chachiensis
Source: Sci Rep. 2020 Apr 8;10:6068. doi: 10.1038/s41598-020-62991-z (PMC7142118; doi:10.1038/s41598-020-62991-z)
Supplement: Supplementary file 1 — Supplementary Information. [file 41598_2020_62991_MOESM1_ESM.pdf]

Distinct microbial communities among different tissues of citrus tree *Citrus reticulata* cv. Chachiensis

Yongxian Wu, Mengqiu Qu, Xinhua Pu, Jintian Lin*, Benshui Shu*

Guangzhou City Key Laboratory of Subtropical Fruit Trees Outbreak Control,
Zhongkai University of Agriculture and Engineering, Guangzhou, China

* Correspondence authors at: Guangzhou City Key Laboratory of Subtropical Fruit Trees Outbreak Control, Institute for Management of Invasive Alien Species, 313 Yingdong teaching building, Zhongkai University of Agriculture and Engineering, Guangzhou, 510225, PR China.

E-mail addersses: linjtian@163.com；shubenshui@126.com

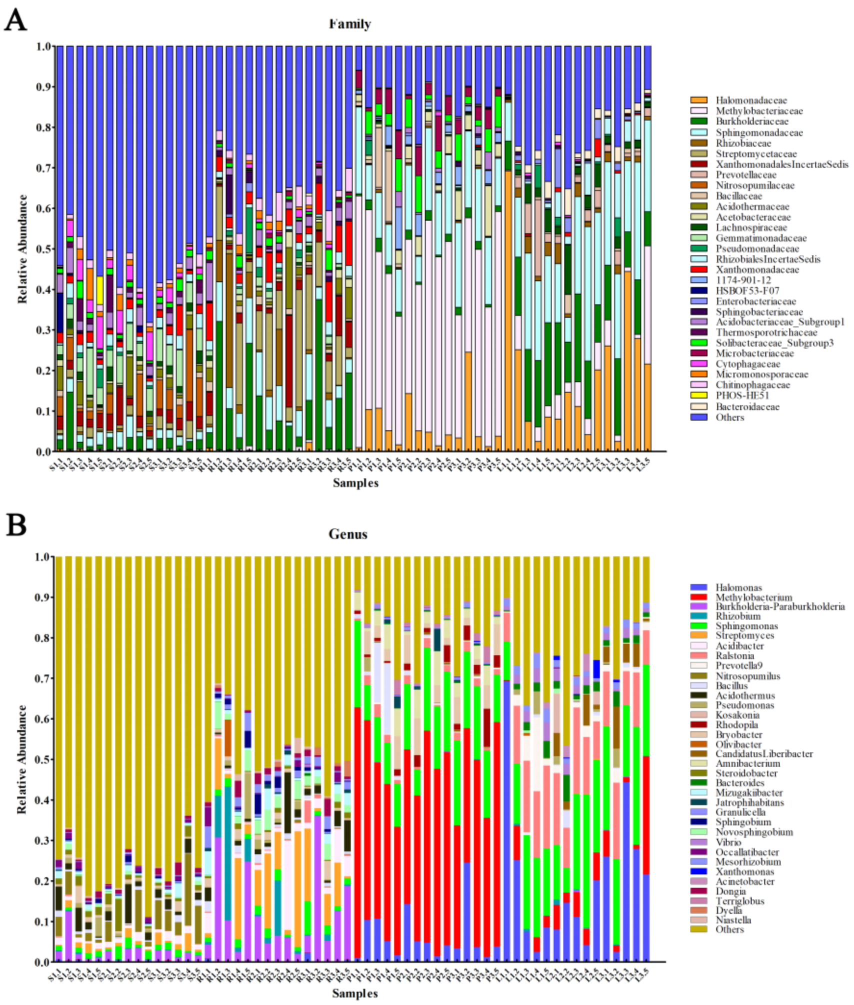

Supplemental Figure 1. The analysis of microbial communities in different tissues of citrus trees at genus and family levels A: The core families of microbial communities in different tissues of citrus trees. B: The core genus of microbial communities in different tissues of citrus trees.

Table S1 Statistics on the number of Tags and OTUs in each sample of 16S

Sample_Name	Total_tag	Taxon_Tag	Unclassified_Mitochondria_ Chloroplast_tag	Unique_Tag	OTU_number
**S1.1**	54752	53167	273	1312	1525
**S1.2**	55359	53800	196	1363	2090
**S1.3**	48242	46456	104	1682	2353
**S1.4**	41239	39581	258	1400	1830
**S1.5**	31666	30182	89	1395	1809
**R1.1**	48095	24190	23399	506	1160
**R1.2**	53683	36210	16993	480	1058
**R1.3**	59333	49630	9239	464	1070
**R1.4**	47804	26602	20840	362	1145
**R1.5**	55665	48111	7024	530	1201
**S2.1**	51674	50638	51	985	1942
**S2.2**	43824	42495	75	1254	1788
**S2.3**	41027	39763	158	1106	1831
**S2.4**	30936	29879	163	894	1682
**S2.5**	32169	31330	47	792	1775
**R2.1**	35226	12429	22465	332	1055
**R2.2**	34661	9460	24953	248	954
**R2.3**	38354	29994	7706	654	1115
**R2.4**	48037	25572	22010	455	944
**R2.5**	38418	24745	13346	327	1211
**S3.1**	50018	48717	105	1196	2210
**S3.2**	42982	40559	838	1585	2316
**S3.3**	41075	39782	302	991	1971
**S3.4**	34598	33895	124	579	1430
**S3.5**	39639	38711	202	726	1779
**R3.1**	49077	30228	18201	648	1345
**R3.2**	33433	16523	16636	274	595
**R3.3**	33378	22063	10873	442	1342
**R3.4**	53774	19804	33693	277	758
**R3.5**	59407	17121	41932	354	767
**P1.1**	30756	5637	25064	55	159
**P1.2**	38542	4667	33786	89	193
**P1.3**	36609	1747	34815	47	165
**P1.4**	37017	4431	32533	53	173
**P1.5**	59724	3655	55999	70	138
**L1.1**	41723	4232	37362	129	122
**L1.2**	39820	223	39524	73	62
**L1.3**	42809	147	42598	64	60
**L1.4**	55464	280	55081	103	62
**L1.5**	33101	749	32255	97	124
**P2.1**	49899	4085	45747	67	161

**P2.2**	43473	5523	37862	88	137
**P2.3**	33843	978	32826	39	98
**P2.4**	32749	2845	29791	113	141
**P2.5**	52053	5443	46529	81	158
**L2.1**	37424	384	36970	70	82
**L2.2**	32586	444	32048	94	119
**L2.3**	32250	905	31232	113	161
**L2.4**	41141	903	40141	97	126
**L2.5**	43436	337	42983	116	70
**P3.1**	36358	2559	33725	74	174
**P3.2**	31252	5877	25303	72	159
**P3.3**	38382	2779	35488	115	161
**P3.4**	41613	7592	33827	194	188
**P3.5**	37415	1251	36086	78	89
**L3.1**	49391	1063	48190	138	118
**L3.2**	41122	319	40718	85	79
**L3.3**	45700	428	45184	88	77
**L3.4**	57763	2508	55096	159	149
**L3.5**	45643	2174	43384	85	145

Table S2 The abundance of species with significant differences between groups

Vs-groups	Taxa	mean(group1)	mean(group2)	p value	q value
**R-vs-P**	*Halomonas*	0.0035	0.063694	0.000999	0.002821
**R-vs-P**	*Methylobacterium*	0.000236	0.426113	0.000999	0.002821
**R-vs-P**	*Burkholderia-Paraburkholderia*	0.11761	0.000199	0.000999	0.002821
**R-vs-P**	*Rhizobium*	0.046036	0.000508	0.000999	0.002821
**R-vs-P**	*Sphingomonas*	0.020822	0.150435	0.000999	0.002821
**R-vs-P**	*Streptomyces*	0.093995	0.000114	0.000999	0.002821
**R-vs-P**	*Acidibacter*	0.035606	2.58E-05	0.000999	0.002821
**R-vs-P**	*Ralstonia*	0.006281	0.016324	0.015984	0.036029
**R-vs-P**	*Acidothermus*	0.027737	5.53E-05	0.000999	0.002821
**R-vs-P**	*Rhodopila*	0	0.022417	0.000999	0.002821
**R-vs-P**	*Bryobacter*	0.012021	0.044356	0.000999	0.002821
**R-vs-P**	*Amnibacterium*	0.000177	0.04069	0.000999	0.002821
**R-vs-P**	*Novosphingobium*	0.028879	0.000136	0.000999	0.002821
**R-vs-P**	*Mizugakiibacter*	0.018977	3.32E-05	0.000999	0.002821
**R-vs-P**	*Sphingobium*	0.012253	0	0.000999	0.002821
**R-vs-P**	*Mesorhizobium*	0.014445	0.002888	0.001998	0.005433
**R-vs-P**	*Dongia*	0.014092	6.63E-05	0.000999	0.002821
**R-vs-P**	*Terriglobus*	2.21E-05	0.010507	0.000999	0.002821
**R-vs-P**	*Rhizomicrobium*	0.015013	1.84E-05	0.000999	0.002821
**R-vs-P**	*Bradyrhizobium*	0.020148	0.001345	0.000999	0.002821
**R-vs-S**	*Burkholderia-Paraburkholderia*	0.11761	0.023375	0.001998	0.015736

**R-vs-S**	*Rhizobium*	0.046036	0.001621	0.000999	0.010189
**R-vs-S**	*Streptomyces*	0.093995	0.01394	0.000999	0.010189
**R-vs-S**	*Nitrosopumilus*	0.000969	0.04888	0.000999	0.010189
**R-vs-S**	*Steroidobacter*	0.005721	0.018188	0.000999	0.010189
**R-vs-S**	*Novosphingobium*	0.028879	0.004616	0.000999	0.010189
**R-vs-S**	*Sphingobium*	0.012253	0.003588	0.033966	0.14376
**R-vs-S**	*Mesorhizobium*	0.014445	0.002034	0.000999	0.010189
**R-vs-S**	*Bradyrhizobium*	0.020148	0.004001	0.000999	0.010189
**R-vs-S**	*Cellulosilyticum*	0.002406	0.014309	0.000999	0.010189
**R-vs-S**	*Gemmatimonas*	0.001768	0.013966	0.000999	0.010189
**R-vs-S**	*Haliangium*	0.003445	0.014456	0.000999	0.010189
**R-vs-L**	*Halomonas*	0.0035	0.195745	0.000999	0.001414
**R-vs-L**	*Methylobacterium*	0.000236	0.053157	0.000999	0.001414
**R-vs-L**	*Burkholderia-Paraburkholderia*	0.11761	0.000586	0.000999	0.001414
**R-vs-L**	*Rhizobium*	0.046036	0.002354	0.002997	0.003989
**R-vs-L**	*Sphingomonas*	0.020822	0.198773	0.000999	0.001414
**R-vs-L**	*Streptomyces*	0.093995	0.000405	0.000999	0.001414
**R-vs-L**	*Acidibacter*	0.035606	0.000453	0.000999	0.001414
**R-vs-L**	*Ralstonia*	0.006281	0.14041	0.000999	0.001414
**R-vs-L**	*Prevotella9*	0.000601	0.029966	0.000999	0.001414
**R-vs-L**	*Acidothermus*	0.027737	0.000136	0.000999	0.001414
**R-vs-L**	*CandidatusLiberibacter*	0.000136	0.027822	0.000999	0.001414
**R-vs-L**	*Bryobacter*	0.012021	2.95E-05	0.000999	0.001414
**R-vs-L**	*Novosphingobium*	0.028879	0.001304	0.000999	0.001414
**R-vs-L**	*Bacteroides*	0.000678	0.022082	0.000999	0.001414
**R-vs-L**	*Mizugakiibacter*	0.018977	0	0.000999	0.001414
**R-vs-L**	*Sphingobium*	0.012253	0.000173	0.000999	0.001414
**R-vs-L**	*Vibrio*	0.000788	0.021474	0.000999	0.001414
**R-vs-L**	*Dongia*	0.014092	7.00E-05	0.000999	0.001414
**R-vs-L**	*Rhizomicrobium*	0.015013	0.000147	0.000999	0.001414
**R-vs-L**	*Ensifer*	0.002409	0.017897	0.000999	0.001414
**R-vs-L**	*Bradyrhizobium*	0.020148	0.011347	0.010989	0.013069
**R-vs-L**	*Bosea*	0.00186	0.016899	0.000999	0.001414
**R-vs-L**	*Ruegeria*	0.000589	0.014751	0.000999	0.001414
**R-vs-L**	*Faecalibacterium*	0.000748	0.012463	0.000999	0.001414
**R-vs-L**	*Cellulophaga*	0.000317	0.010636	0.000999	0.001414
**P-vs-S**	*Halomonas*	0.063694	0.003025	0.000999	0.001028
**P-vs-S**	*Methylobacterium*	0.426113	0.000313	0.000999	0.001028
**P-vs-S**	*Burkholderia-Paraburkholderia*	0.000199	0.023375	0.000999	0.001028
**P-vs-S**	*Sphingomonas*	0.150435	0.014003	0.000999	0.001028
**P-vs-S**	*Streptomyces*	0.000114	0.01394	0.000999	0.001028
**P-vs-S**	*Acidibacter*	2.58E-05	0.01359	0.000999	0.001028
**P-vs-S**	*Ralstonia*	0.016324	0.000674	0.000999	0.001028
**P-vs-S**	*Nitrosopumilus*	4.79E-05	0.04888	0.000999	0.001028

**P-vs-S**	*Acidothermus*	5.53E-05	0.027431	0.000999	0.001028
**P-vs-S**	*Rhodopila*	0.022417	0	0.000999	0.001028
**P-vs-S**	*Bryobacter*	0.044356	0.009354	0.000999	0.001028
**P-vs-S**	*Amnibacterium*	0.04069	0.000111	0.000999	0.001028
**P-vs-S**	*Steroidobacter*	2.58E-05	0.018188	0.000999	0.001028
**P-vs-S**	*Terriglobus*	0.010507	2.58E-05	0.000999	0.001028
**P-vs-S**	*Rhizomicrobium*	1.84E-05	0.01978	0.000999	0.001028
**P-vs-S**	*Cellulosilyticum*	0	0.014309	0.000999	0.001028
**P-vs-S**	*Gemmatimonas*	0.00014	0.013966	0.000999	0.001028
**P-vs-S**	*Haliangium*	0	0.014456	0.000999	0.001028
**P-vs-L**	*Halomonas*	0.063694	0.195745	0.004995	0.001099
**P-vs-L**	*Methylobacterium*	0.426113	0.053157	0.000999	0.000255
**P-vs-L**	*Sphingomonas*	0.150435	0.198773	0.042957	0.006603
**P-vs-L**	*Ralstonia*	0.016324	0.14041	0.000999	0.000255
**P-vs-L**	*Prevotella9*	0.001684	0.029966	0.000999	0.000255
**P-vs-L**	*CandidatusLiberibacter*	0.00137	0.027822	0.000999	0.000255
**P-vs-L**	*Rhodopila*	0.022417	0	0.000999	0.000255
**P-vs-L**	*Bryobacter*	0.044356	2.95E-05	0.000999	0.000255
**P-vs-L**	*Amnibacterium*	0.04069	0.000884	0.000999	0.000255
**P-vs-L**	*Bacteroides*	0.001706	0.022082	0.000999	0.000255
**P-vs-L**	*Vibrio*	0.002269	0.021474	0.000999	0.000255
**P-vs-L**	*Mesorhizobium*	0.002888	0.018266	0.000999	0.000255
**P-vs-L**	*Terriglobus*	0.010507	0	0.000999	0.000255
**P-vs-L**	*Ensifer*	0.002431	0.017897	0.000999	0.000255
**P-vs-L**	*Bradyrhizobium*	0.001345	0.011347	0.000999	0.000255
**P-vs-L**	*Bosea*	0.00228	0.016899	0.000999	0.000255
**P-vs-L**	*Ruegeria*	0.001754	0.014751	0.000999	0.000255
**P-vs-L**	*Faecalibacterium*	0.001367	0.012463	0.000999	0.000255
**P-vs-L**	*Cellulophaga*	0.001127	0.010636	0.000999	0.000255
**S-vs-L**	*Halomonas*	0.003025	0.195745	0.000999	0.000804
**S-vs-L**	*Methylobacterium*	0.000313	0.053157	0.000999	0.000804
**S-vs-L**	*Burkholderia-Paraburkholderia*	0.023375	0.000586	0.000999	0.000804
**S-vs-L**	*Sphingomonas*	0.014003	0.198773	0.000999	0.000804
**S-vs-L**	*Streptomyces*	0.01394	0.000405	0.000999	0.000804
**S-vs-L**	*Acidibacter*	0.01359	0.000453	0.000999	0.000804
**S-vs-L**	*Ralstonia*	0.000674	0.14041	0.000999	0.000804
**S-vs-L**	*Prevotella9*	0.000147	0.029966	0.000999	0.000804
**S-vs-L**	*Nitrosopumilus*	0.04888	0	0.000999	0.000804
**S-vs-L**	*Acidothermus*	0.027431	0.000136	0.000999	0.000804
**S-vs-L**	*CandidatusLiberibacter*	0	0.027822	0.000999	0.000804
**S-vs-L**	*Steroidobacter*	0.018188	0	0.000999	0.000804
**S-vs-L**	*Bacteroides*	0.000162	0.022082	0.000999	0.000804
**S-vs-L**	*Vibrio*	0.00014	0.021474	0.000999	0.000804
**S-vs-L**	*Mesorhizobium*	0.002034	0.018266	0.000999	0.000804

**S-vs-L**	*Rhizomicrobium*	0.01978	0.000147	0.000999	0.000804
**S-vs-L**	*Ensifer*	0.000612	0.017897	0.000999	0.000804
**S-vs-L**	*Bradyrhizobium*	0.004001	0.011347	0.001998	0.001538
**S-vs-L**	*Cellulosilyticum*	0.014309	0	0.000999	0.000804
**S-vs-L**	*Bosea*	0.000354	0.016899	0.000999	0.000804
**S-vs-L**	*Gemmatimonas*	0.013966	0.000225	0.000999	0.000804
**S-vs-L**	*Ruegeria*	7.37E-05	0.014751	0.000999	0.000804
**S-vs-L**	*Faecalibacterium*	0.000122	0.012463	0.000999	0.000804
**S-vs-L**	*Haliangium*	0.014456	9.95E-05	0.000999	0.000804
**S-vs-L**	*Cellulophaga*	5.53E-05	0.010636	0.000999	0.000804

R: Roots, S: Soils, P: Phloem, L: Leaves
